# Supplementary material for: Disentangling metabolic functions of bacteria in the honey bee gut
Source: PLoS Biol. 2017 Dec 12;15(12):e2003467. doi: 10.1371/journal.pbio.2003467 (PMC5726620; doi:10.1371/journal.pbio.2003467)
Supplement: S8 Data — (ZIP) [file pbio.2003467.s008.zip › Z-score_plots/855 microbial substrate Quercetin 3-O-glucoside.pdf]

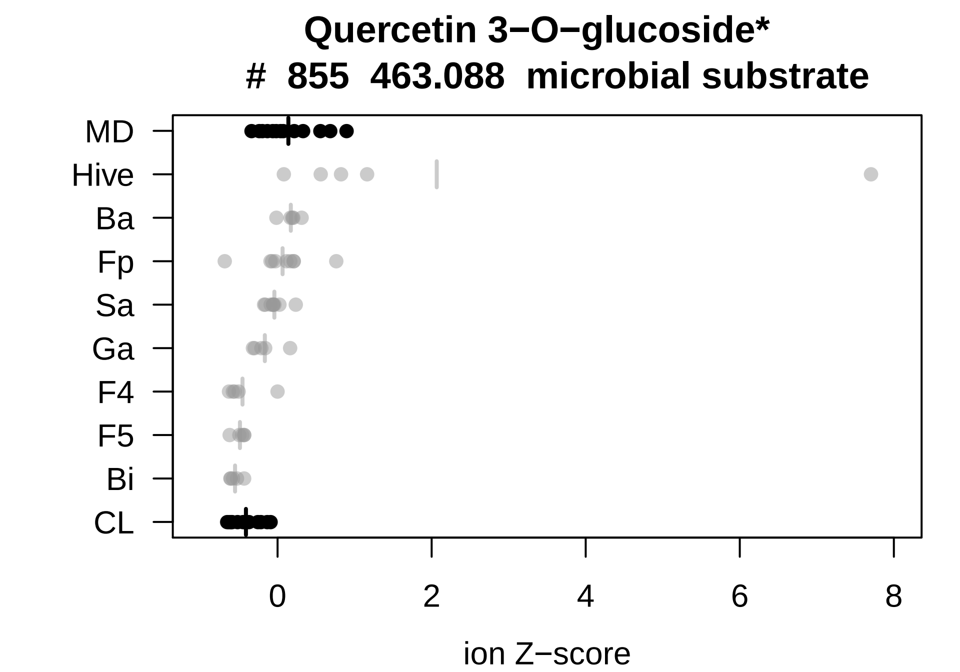
Quercetin 3−O−glucoside*
855 463.088 microbial substrate
MD
Hive
Ba
Fp
Sa
Ga
F4
F5
Bi
CL
0
2
4
6
8
ion Z−score
